# Novel information on the cranial anatomy of the tapejarine pterosaur *Caiuajara dobruskii*

**DOI:** 10.1371/journal.pone.0277780

**Published:** 2022-12-15

**Authors:** Lucas Canejo, Borja Holgado, Luiz C. Weinschütz, João H. Z. Ricetti, Everton Wilner, Alexander W. A. Kellner

**Affiliations:** 1 Laboratory of Systematics and Taphonomy of Fossil Vertebrates, Departamento de Geologia e Paleontologia, Museu Nacional/Universidade Federal do Rio de Janeiro, Rio de Janeiro, RJ, Brazil; 2 Institut Català de Paleontologia Miquel Crusafont, Universitat Autònoma de Barcelona, Catalonia, Spain; 3 Centro de Pesquisa Paleontológica, Universidade do Contestado, Mafra, SC, Brazil; University College London, UNITED KINGDOM

## Abstract

*Caiuajara dobruskii* is a tapejarid pterosaur from the Cretaceous of the ‘Cemitério dos Pterossauros’ (pterosaur graveyard) site, a unique pterosaur bonebed which is located at the municipality of Cruzeiro do Oeste (Paraná, Brazil). Preliminary inferences on *Caiuajara* morphology were founded on a few partial skeletons, with no detail on the skull anatomy. Here we describe a new specimen from the pterosaur graveyard site, which corresponds to the most complete skull of *Caiuajara dobruskii* known so far. Furthermore, we describe and compare other specimens including the holotype, a paratype, and several other undescribed specimens. The new specimen preserves the posterior portion of the skull, allowing a better comprehension of its morphology and provides an appreciation of the anatomic structures of the basicranium, enabling better interpretation of this region. We also described the lower jaw of *Caiuajara*, reporting a unique feature of its symphyseal which adds to the diagnosis for the species. A variability in the premaxillary crest is also noted in different specimens of *Caiuajara*, which might be interpreted as sexual dimorphism or ontogenetic variability. Therefore, those new findings allow a better comprehension of its skull and enables a more precise comparison between the skulls of those extinct flying reptiles.

## Introduction

Pterosaurs were the first vertebrates to acquire active flight and lived during most of the Mesozoic Era, with their latest record dated to 66 million years ago [1–7]. Notwithstanding their remains are found in all continents [8–10], the pterosaur fossil record is frequently limited to fragmentary and isolated individuals with occasional biased stratigraphic control [5, 8, 11, 12]. This has caused several taxonomic controversies, with the establishment of several ambiguous taxa [12–16], and debatable proposals about their ontogeny and intraspecific variability [12, 17–20]. The only cases in the fossil record of unquestionable ontogenetic series of pterosaurs are scant [21–26], of which most of these are found in bonebeds. Only three occurrences of pterosaur bonebeds have been reported: in Argentina (San Luis Province), the ctenochasmatid *Pterodaustro guinazui* was found in the outcrops of the Albian Lagarcito Formation [22, 23, 27, 28]; in Northwestern China (Xingjiang Autonomous Region), the anhanguerian *Hamipterus tianshanensis* [25, 26] was recovered in outcrops of the Lower Cretaceous Tugulu Group; and in Southern Brazil (Paraná), the tapejarine *Caiuajara dobruskii* was found in the mid-to-late Cretaceous ‘Cemitério dos pterossauros’ (pterosaur graveyard) site of the municipality of Cruzeiro do Oeste, state of Paraná [24].

The sole Brazilian site of ‘Cemitério dos pterossauros’ is the only bone bed known so far where more than one pterosaur species was found: the already mentioned *Caiuajara dobruskii* Manzig et al. 2014, which is the predominant species in the vertebrate assemblage; and *Keresdrakon vilsoni* Kellner et al. 2019 [29]. Dinosaurs were also present in these outcrops, represented by two known species: *Vespersaurus paranaensis* Langer et al. 2019 [30] and *Berthasaura leopoldinae* Souza et al. 2021 [31] together with the squamate lepidosaur *Gueragama sulamericana* Simões et al. 2015 [32]. No remains of eggs or soft tissue, occasionally recorded in other deposits (e.g., [25, 33]) have been reported so far. The ‘Cemitério dos pterossauros’ site comprises a sequence of aeolian sandstones in to the Caiuá Group and, together with the sediments of the Bauru Group, represent an ancient desert that covered part of southern Brazil in the Cretaceous period.

**Fig 1:**
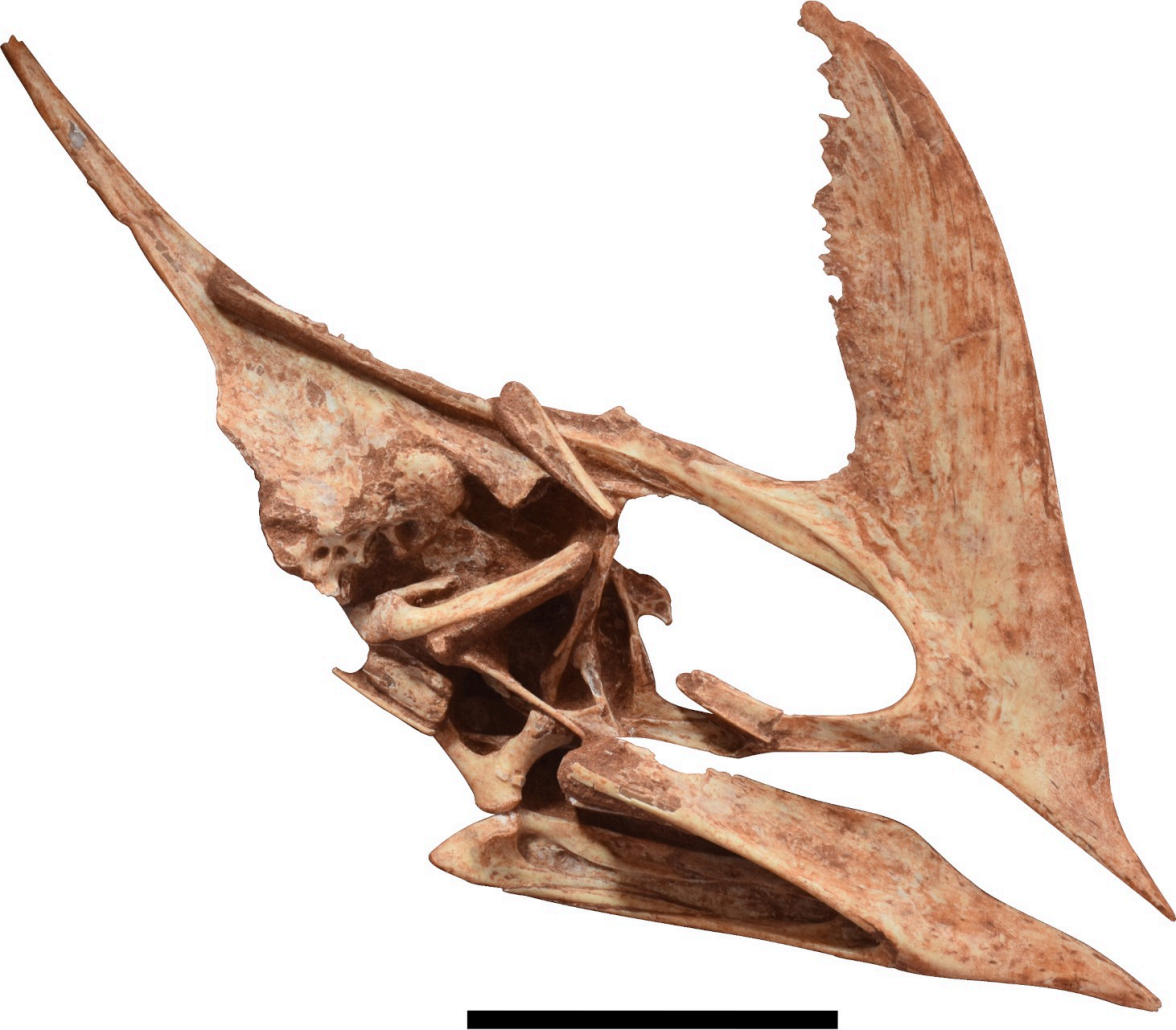
Photograph of CP.V 8175 in right lateral view, a new specimen identified as *Caiuajara dobruskii*. Scale bar equals 50 mm.

The huge amount of pterosaur specimens belonging to *Caiuajara* displays a high morphological variability, both in size and shape. Some of such traits were originally pointed out as ontogenetic features, like the size and inclination of the sagittal crest [24], referring to most of the individuals as juvenile and subadults. However, its original description does not mention the bones forming the posterior portion of the skull due the lack of preservation. In this sense, the need for anatomical information for this area of the skull remains unsolved to date. In this work, we describe a nearly complete skull, which represents the best-preserved skull of *Caiuajara dobruskii* known so far (Fig 1). In addition to this, we reassess some of the previously described specimens and compare them with the new ones undescribed so far, allowing both a revision of the original diagnosis of *Caiuajara dobruskii* as well elucidating the variability in the cranial morphology.

## Material and methods

### Material

The specimen CP.V 8175 was collected in 2014, at the ‘Cemitério dos pterossauros’ site and it is housed at the collection of the Centro de Pesquisa Paleontológica, Universidade do Contestado (CENPALEO). Together with the new specimen which constitutes the focus of our results (CP.V 8175), we also analysed the specimens of the *Caiuajara dobruskii* type series (CP.V 1005; CP.V 1050–1 and CP.V 1449) as well as other not previously described specimens (CP.V 1448; CP.V 5703; and CP.V 8171). No permits were required for the described study, which complied with all relevant regulations.

CP.V 8175 was mechanically prepared up until it was revealed a partially articulated skull and lower jaw exposed on the right side, with the occlusal region opened. The material is associated with some postcranial elements as a left pteroid, left fused distal and proximal carpals, left metacarpals (I to IV), an isolated manual phalanx, and an ungueal phalanx (S1 and S2 Figs). The bones are three dimensionally preserved and still partially confined in its matrix. Incipient weathering occurs all over the cranial remains, especially on the frontoparietal surface. The majority of the compression occurs over the lower jaw, so the right dentary ramus was cracked and displaced medially. Most of the elements surrounding the orbit are disarticulated and partially eroded. The anatomical nomenclature follows that of Romer [34]. Anatomical comparisons were made between the aforementioned new specimen, most of the previously described specimens, and other new specimens with cranial remains. See Table 1 for further details on the sample studied. Pictures were made using a Canon Sl3 equipped with Ef100 f2.8 macro lenses and edited using GIMP editor. The software GIMP was used for the silhouette drawings, whilst the software ImageJ [35] was used for specimen measurements.

**Table 1:** *Caiuajara dobruskii* analysed specimens with listed content.

Specimen	Type	Content
CP.V 8175	New specimen	Partially articulated skull and lower jaw, a left pteroid, left fused distal and proximal carpals, left metacarpals I to IV, an isolated phalanx, and ungueal phalanx.
CP.V 1449	Holotype (Manzig et al., 2014)	A fairly complete fractured skull; a partial lower jaw; an isolated mid-cervical vertebrae; right and left distal carpal series; right pteroid, right(?) metacarpals I-III, a right metacarpal IV, two right first phalanges of the manual digit IV, a left first phalanx of the manual digit IV, a right second phalanx of the manual digit IV, a second phalanx of the manual digit IV, and a fourth phalanx of the manual digit IV.
CP.V 8171	New specimen	Two premaxillomaxillaries.
CP.V 5703	Paratype (Manzig et al., 2014)	A skull and lower jaw, four cervical vertebrae, right and left metacarpal IV, left first phalanx of manual digit IV.
CP.V 1005	Paratype (Manzig et al., 2014)	A partial skull, including an almost complete rostrum, and lower jaw.
CP.V 1448	New specimen	A skull, including an almost complete rostrum, and the anterior portion of the nasoantorbital fenestra.
CP.V 1050–1	Referred specimen (Manzig et al., 2014)	A block including a several small individuals, including cranial and postcranial elements.

**Fig 2:**
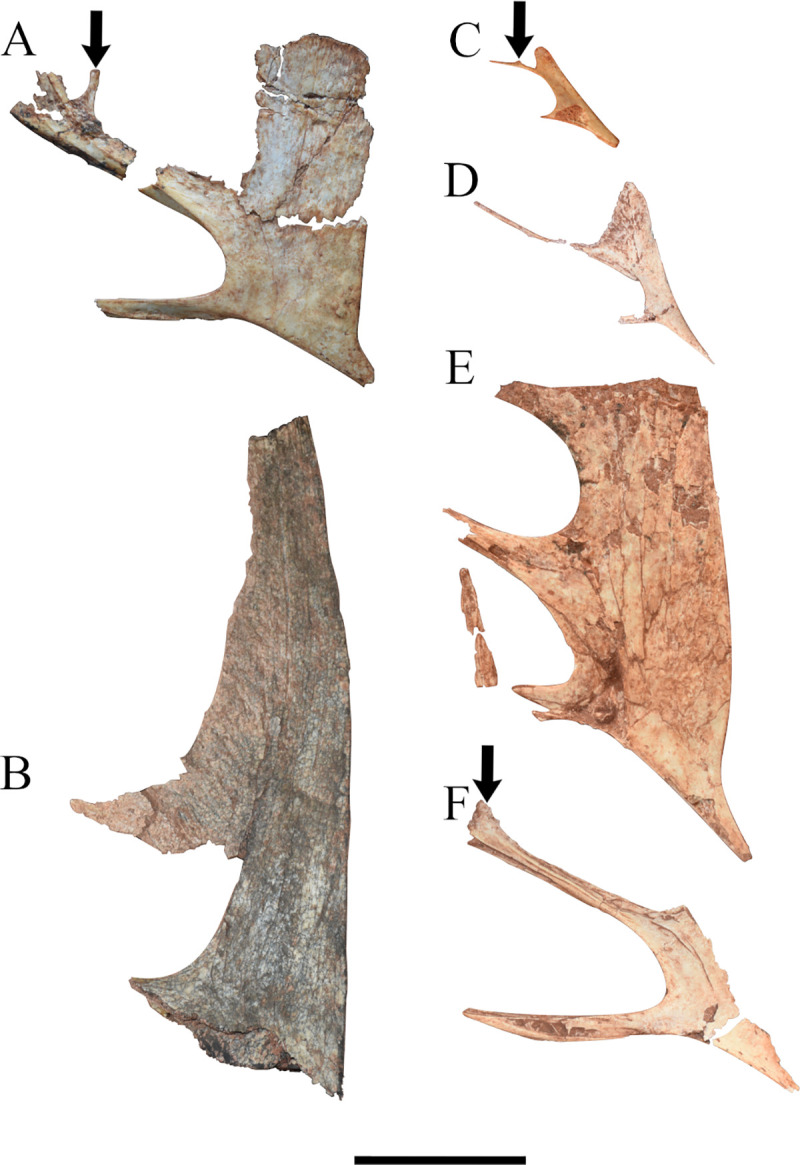
Picture of skulls belonging to several specimens of *Caiuajara dobruskii*. (A) specimen CP.V 1449 (holotype) in right lateral view; (B) specimen CP.V 1005; (C) specimen CP.V 1050–1; (D) inverted specimen CP.V 8171 in left lateral view; (E) inverted specimen CP.V 1448; (F) inverted specimen CP.V 5703. Bold arrows indicate the slight dorsal expansion of the premaxillary posterior process. Scale bar equals 50 mm.

**Fig 3:**
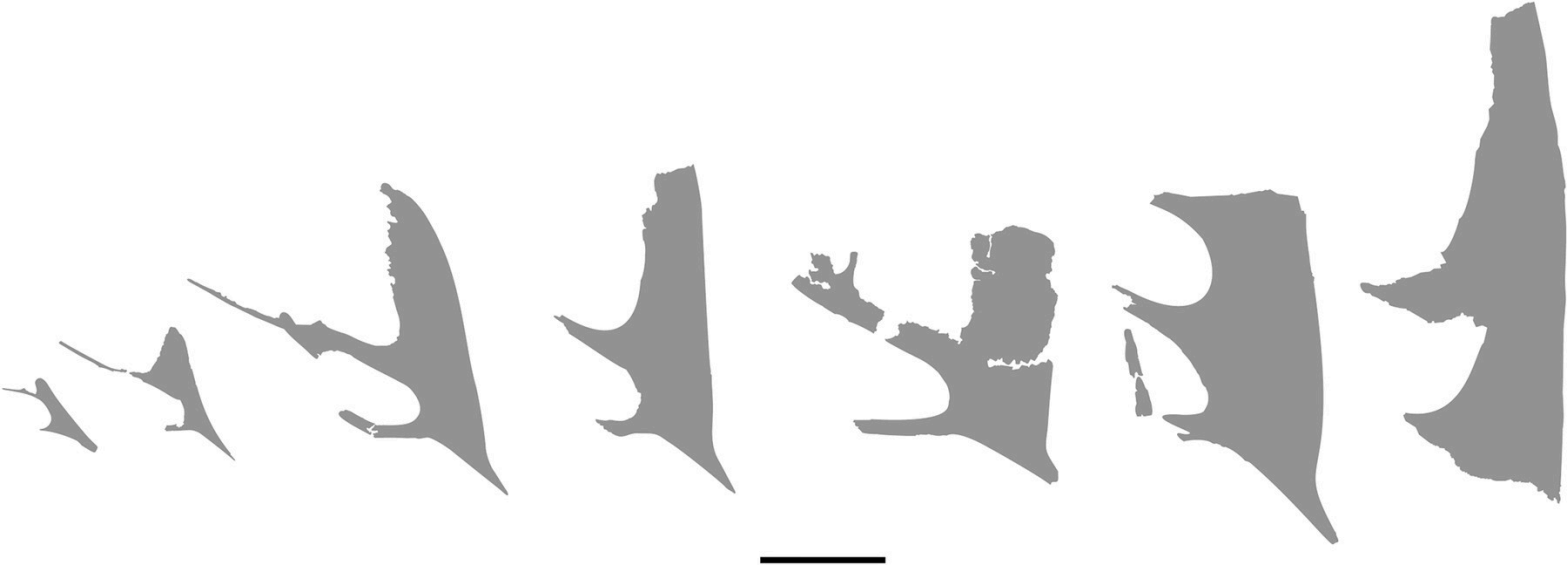
Morphological disparity in some *Caiuajara dobruskii* from smaller to larger individuals. From left to right, CP.V 1050–1, CP.V 8171, CP.V 8175, CP.V 1447, CP.V 1449, CP. 1448, and CP.V 1005. Scale bar equals 50 mm.

In order to increase the understanding of the cranial anatomy of *Caiuajara dobruskii*, we describe other specimens, mainly to describe and compare anatomical features not preserved in the specimen CP.V 8175 and/or to confirm uncertain morphologies. The specimens analysed here are the following: the holotype (CP.V 1449) (Fig 2A), paratype CP.V 1005 (Fig 2B), and the previously described specimen CP.V 1050–1 (Fig 2C), but also the undescribed specimens, CP.V 8171 (Fig 2D), CP.V 1448 (Fig 2E) and CP.V 5703 (Fig 2F) (See Fig 3 for size comparison). Henceforth, we describe such features on each anatomical region of the cranial skeleton taking into account all specimens aforementioned.

### Anatomical abbreviations

afs, adductor fossa; am, adductor mandibulae; ang, angular; art, articular; bo, basioccipital; bs, basisphenoid; d dentary; dcr, dentary crest; exo, exoccipital; expv, ventral expansion of premaxilla; expd, dorsal expansion of premaxilla; fom, foramen magnum; fopn, foramen pneumaticum; f, frontal; hy hyoid bone; j, jugal; la, lacrimal; m, maxilla; mansy, mandibular symphysis; manr, mandibular ramus; mcfs, meckelian fossa; n, nasal; o, occipital region; oc, occipital condyle; op, opisthotic; p, parietal; pcfe, postcranial fenestra; pcr, parietal crest; pm, premaxilla; pmcr, premaxillary crest; pri, palatal ridge; psm, pseudomesethmoid; ptfe, post temporal fenestra; pty, pterygoid; q, quadrate; rapr, retroarticular process; san, surangular; soc, supraoccipital; sq, squamosal; sysh, symphyseal shelf; roman numerals, foramina for cranial nerves; vpt, ventral protrusion.

### Institutional abbreviations

AMNH, American Museum of Natural History (New York, USA); BXGM, Benxi Geological Museum (Liaoning Province, China); CENPALEO, Centro de pesquisa paleontológica da Universidade do Contestado (Mafra, Santa Catarina, Brazil); CP.V, Contestado University *Centro de Pesquisa Paleontológica* vertebrate collection (Mafra, Santa Catarina, Brazil); GP/2E, Laboratório de Paleontologia Sistemática do Instituto de Geociências da Universidade de São Paulo (São Paulo, Brazil); IVPP, Institute of Vertebrate Paleontology and Paleoanthropology (Beijing, China); MCT, *Museu de Ciências da Terra*, Brazilian Geological Survey and *Agência Nacional de Mineração* (Rio de Janeiro, Brazil); MN, Museu Nacional, Universidade Federal do Rio de Janeiro (Rio de Janeiro, Brazil); MPSC, Museu de Paleontologia Plácido Cidade Nuvens de Santana do Cariri (Santana do Cariri, Ceará Brazil); SMNK, Staatliches Museum für Naturkunde Karlsruhe (Karlsruhe, Germany).

## Results

### Systematic paleontology

PTEROSAURIA Kaup, 1834 [36]

PTERODACTYLOIDEA Plieninger, 1901 [37]

TAPEJARIDAE Kellner, 1989 [38]

TAPEJARINAE Kellner, 1989 [38] *sensu* Kellner & Campos, 2007 [39]

TAPEJARINI Andres et al., 2014 [40]

*Caiuajara* Manzig et al., 2014 [24]

*Caiuajara dobruskii* Manzig et al., 2014 [24]

### Emended diagnosis

*Caiuajara dobruskii* can be distinguished from other Tapejarini by the following autapomorphies and exclusive combination of characters: 1) premaxillary anterior end strongly deflected ventrally (~142–149°) relative to upper jaw ventral margin; 2) ventral expansion of the premaxillae projected inside the nasoantorbital fenestra; 3) dentary occlusal surface with a rounded depression; 4) quadrate with an elongated groove on its anterolateral margin; 5) maxilla with a marked lateral depression ventral to anterior portion of the nasoantorbital fenestra; [24]; 6) dentaries merge at three different points, one taking place dorsally in the occlusal region, other ventrally in the dentary crest, and the last between those two, ensuing two opening internally in the symphyseal forming of a symphysis shelf.

### Specimens

#### Holotype

CP.V 1449: Skull and lower jaw with some postcranial elements including an isolated cervical vertebra, dorsal vertebrae and wing elements. Housed at CENPALEO.

#### Referred specimens

CP.V 8175: Skull, lower jaw, proximal epiphysis of left metacarpals I-IV, fused left distal and proximal carpals and pteroid, an isolated phalanx, and ungual phalanx; CP.V 1005, incomplete skull with an elongated crest and the lower jaw; CP.V 1448, partially preserved skull with an expanded sagittal crest; CP.V 5703, skull and lower jaw, four cervical vertebrae, and some elements of the wing; CP.V 8171, partially preserved skull with the posterior process of premaxilla preserved.

### Description

#### Generalities

**Fig 4:**
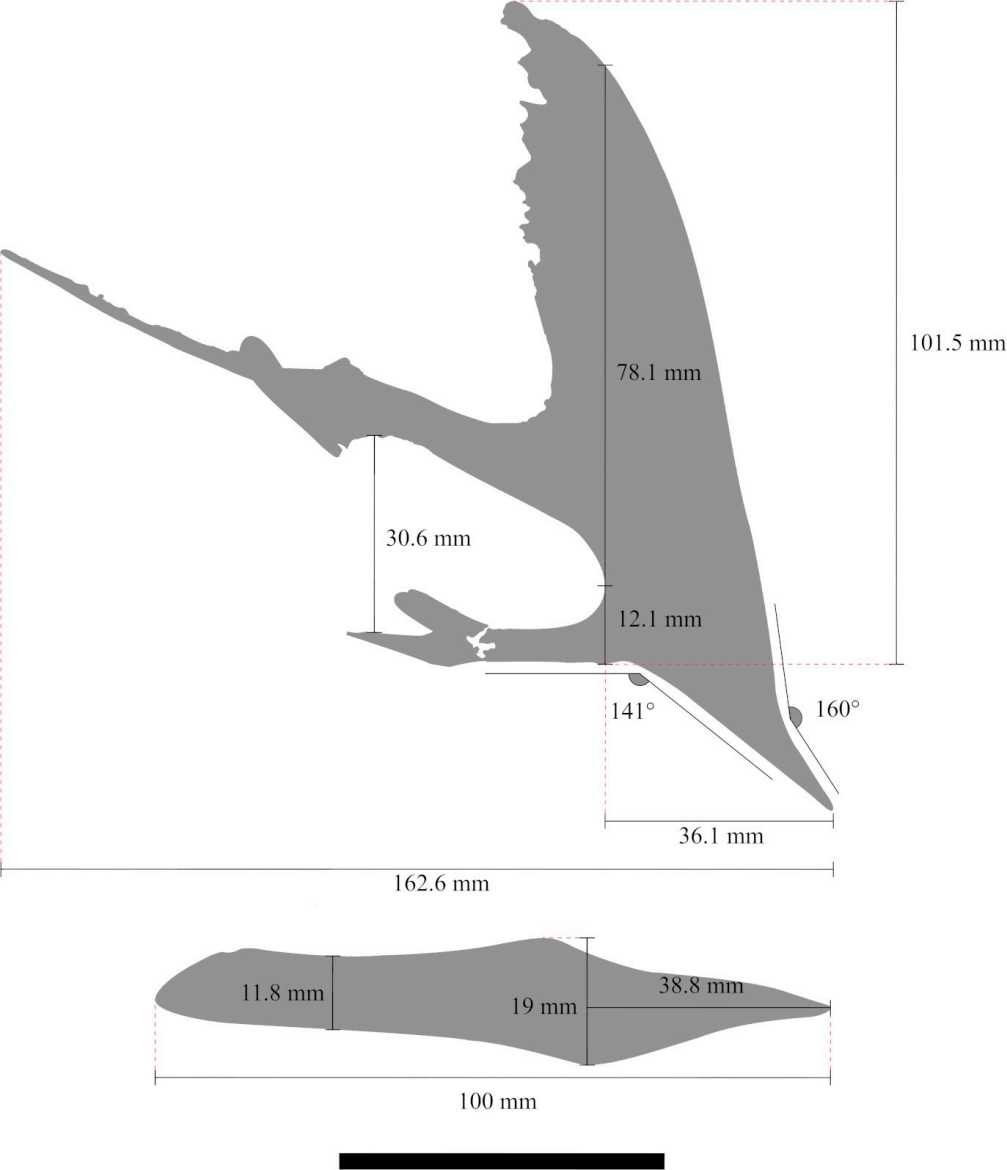
Schematic drawing of specimen CP.V 8175 skull and lower jaw with their respective measures. Scale bar equals 50 mm.

The specimen is composed of a partially articulated skull and lower jaw (Fig 1) exposed in its right lateral profile. It is associated with few postcranial elements as a left pteroid, left fused distal and proximal carpals, left metacarpals I to IV, an isolated phalanx, and ungueal phalanx (Table 1). The skull is mostly weathered in its posterior portion so the parietal process and the occiput are slightly dislocated dorsally. The bones that surround the orbit are mostly disarticulated. Compression happens mostly over the mandible so the right ramus is broken and displaced posteriorly. It presents a blade-like and steeped sagittal crest. Such a feature point to a subadult individual which, whether these associated fused carpals belonging to the same specimen, strengthens that ontogenetic stage. The specimen presents a total length of 162.6 mm measured from the tip of premaxilla to the most posterior level of the parietal process and a maximum height of 101.5 mm measured from the highest point of the sagittal crest to the base of the skull (Table 2; Fig 4). Each portion and relevant bones forming the skull and mandible are described in detail below.

**Table 2:** *Caiuajara dobruskii* CP.V 8175 preserved cranium and postcranial elements measurements.

Element	Extent	Value
**cra**	MHSC	101.5 mm
**cra**	MLC	162.6
**ros**	LPMN	36.1 mm
**ros**	HN	12.1 mm
**ros**	SRDC	141°
**ros**	RV	2.98
**ros**	RI	0.46
**pmcr**	ASCA	160°
**pmcr**	HSCN	78.1 mm
**naofe**	NHP	30.6 mm
**man**	ML	100 mm
**man**	ML/SL	2.6
**d**	DCH	19 mm
**d**	MRH	11.8 mm
**d**	DCH/MRH	1.6
**symph**	SL	38.8 mm
**postcra**	IV METCARP	133.9 mm
**postcra**	PRT	35.7 mm
**postcra**	PH	14.36 mm
**postcra**	UNPH	18.3 mm

Abbreviations of the applied measurements: ASCA, Angulation of the sagittal crest anterior margin; DCH, Dentary crest Height; DCH/MRH, Dentary crest height / Mandibular ramus height; HSCN, Height of the sagittal crest anterior to nasoantorbital fenestra; ML, Mandibular length; MLC, Maximum length of the cranium; LPMN, Length from tip of premaxilla to anterior of nasoantorbital fenestra; METCARP, preserved metacarpal; MHSC, Maximum height from dorsalmost tip of sagittal crest to ventral margin of the skull; MRH, Mandibular ramus height; HN, Height of the anteriormost point of the nasoantorbital fenestra; NHP, Nasoantorbital fenestra highest point; PH, phalanx; PTR, pteroid; RV, Rostral value sensu Kellner 2010; RI, Rostral index sensu Martill & Naish, 2006; SL, Symphysis length; SRDC, Slope of the rostrum downward curvature; UNPH, ungueal phalanx. All anatomical abbreviations are mentioned above.

#### Rostrum

**Fig 5:**
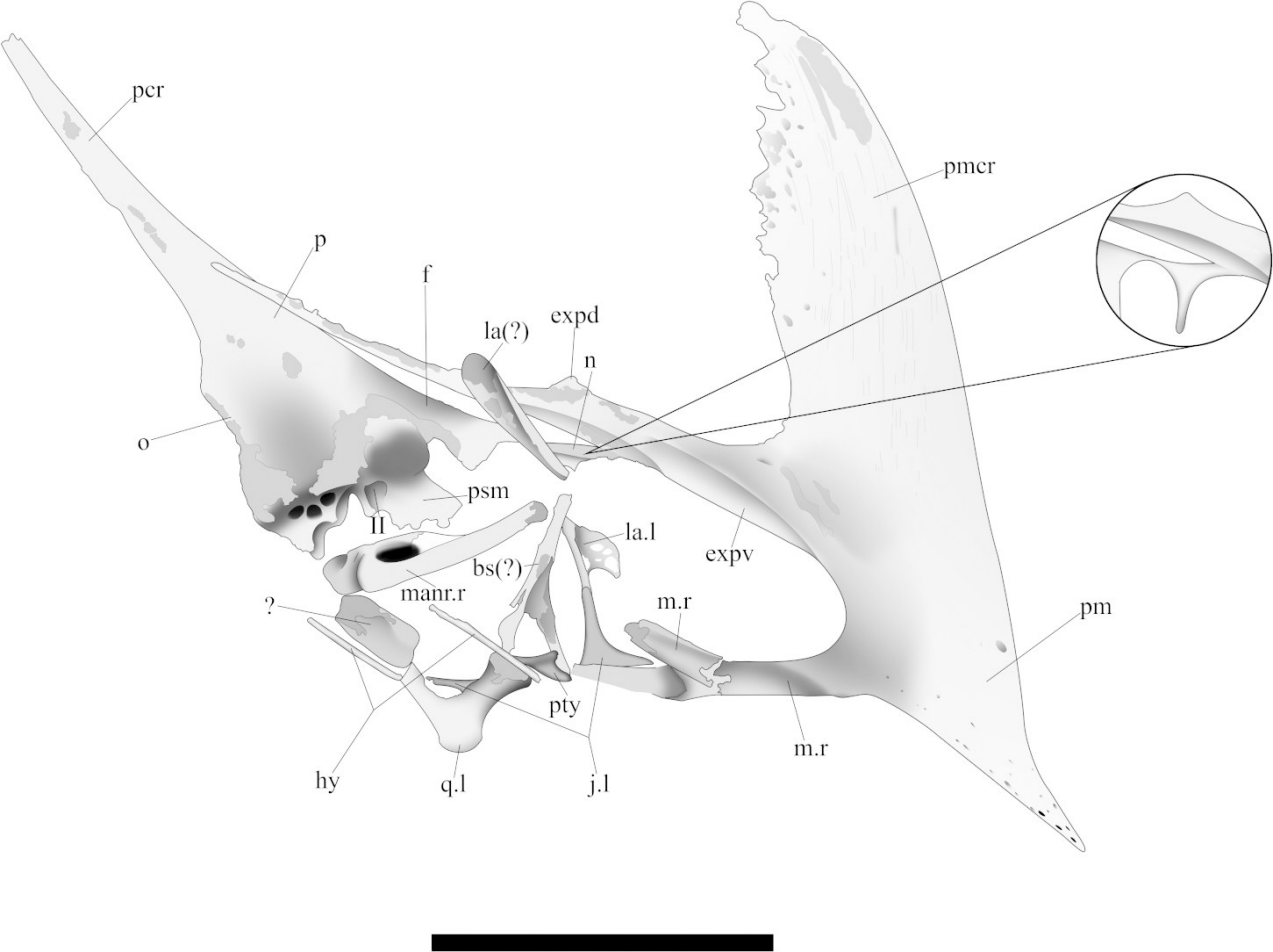
Schematic drawing of CP.V 8175 in right lateral view, a new specimen identified as *Caiuajara dobruskii*. The interpretation of the covered free nasal process is pointed out in detail. Scale bar equals 50 mm.

The maxilla and premaxilla are fused forming a rostrum with no visible suture. The anterior end of the rostrum is inclined downwards, with a slope of 141°. The premaxillary sagittal crest has a thin posterodorsal margin, which is dorsally extended and sub vertically oriented (Fig 5). It bears a striped groove pattern on its lateral surface. The Rostral Value LPMN/HN (*sensu* [41]) is 2.98, whilst the Rostral Index LPMN/HSCN (*sensu* [42]) is 0.46. See Table 2 for further details in these measurements and ratios.

The premaxilla is articulated to the nasal but does not articulate with the dorsal margin of the skull. The premaxillary posterior process is extended further posteriorly, much more than the premaxillary sagittal crest extends dorsally. This posterior process also shows a slight dorsal projection in its middle portion, which tapers abruptly. The right maxilla is broken and slightly displaced, whilst the left one is preserved but partially covered. The maxilla encloses a depression following its ventral margin, creating a sharped ridge laterally to the palatal plate of maxillae.

**Fig 6:**
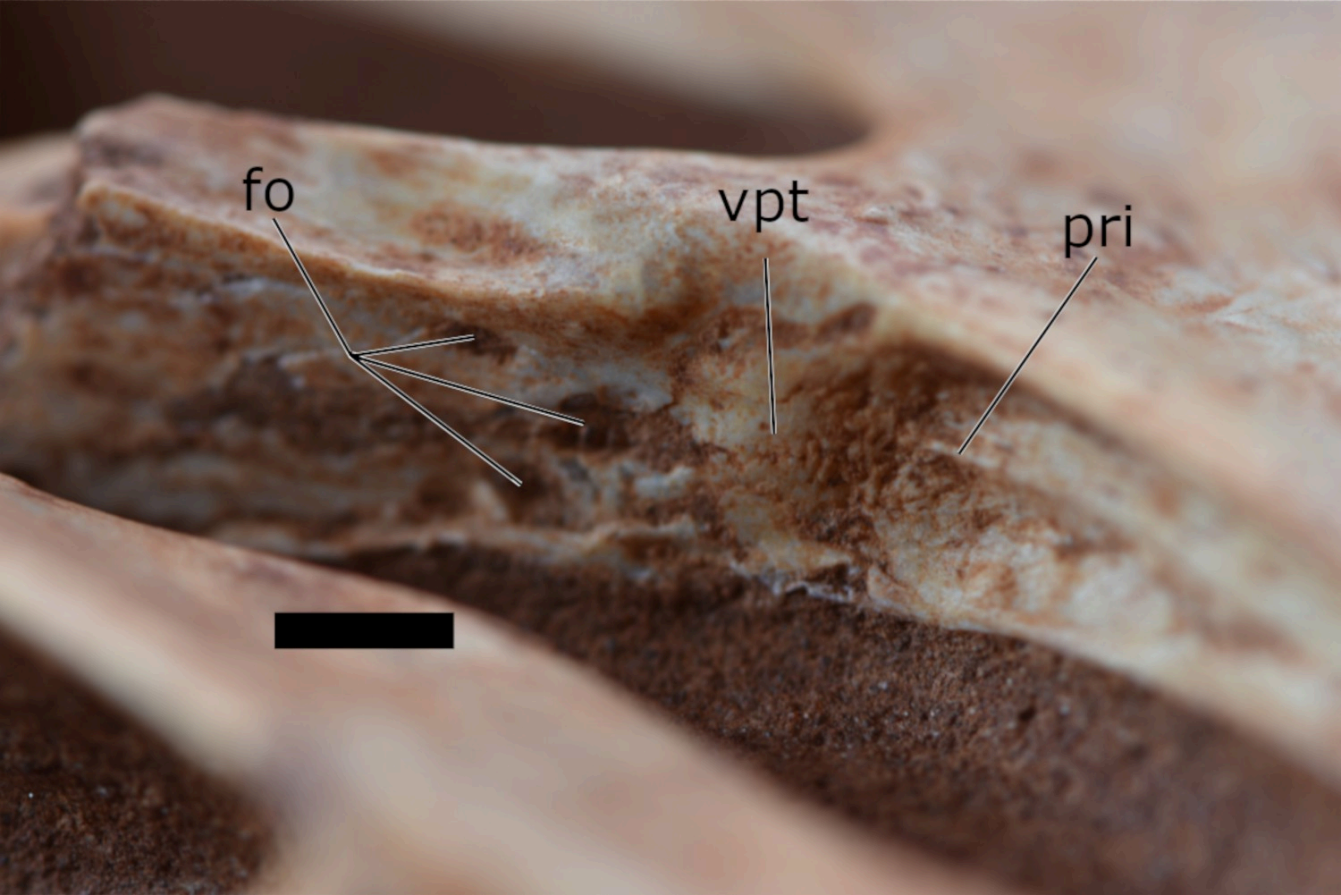
Detailed picture of the palatal region of the specimen CP.V 8175 in ventrolateral view. Scale bar equals 5 mm.

The palate is thinner anteriorly and increases in width posteriorly. In ventral view, the palatal region of the rostrum is pierced by many small pneumatic foramina and bears a regular palatal ridge, which is anteroposteriorly oriented. Posterior to the palatal ridge, the palatal plates of the maxillae develop two small ventral extended protrusions (Fig 6). Posterior to these protrusions, the palate is perforated by three foramina that are quite a bit larger than the anterior ones. The most posterior portion of the palate is missing, preserving neither the palatines nor the vomers.

The nasals are partially preserved, with the anterior and dorsal portions mostly recognizable. The posterior portion is displaced dorsally relative to the anterior portion. The nasal is not articulated with the lacrimal. The anterior tip of the nasal is articulated with the premaxilla. Its middle portion is partially covered by the matrix and what could be the basisphenoid. The nasal in *Caiuajara* is a triangular, curved and discreet developed element forming the dorsoposterior margin of the nasoantorbital fenestra. A thin, free descending process projects from the anterior half of the nasal. That process is covered by what seems to be the basisphenoid (Fig 5). This process bows posteriorly, forming an arch, and ventrally protrudes about 9.4 mm toward the nasoantorbital fenestra. Posteriorly, the nasals articulate with the frontal with an almost vertically and smooth suture separating those bones.

#### Frontal

The ventrolateral portion of the frontal is weathered, exposing the braincase laterally. The frontals cover most of the skull surface over the orbit region. The maximum width is located posteriorly above the orbit. The suture separating the frontal and parietal runs almost vertically, posteriorly to the braincase and reaching the temporal region. The right postorbital process of the frontal is missing.

#### Parietal

The parietal crest is dorsally displaced relative to the premaxilla. The parietal process extends more posteriorly than the premaxilla, tapering at the end (Fig 5). It comprises one-third of the skull’s total length.

#### Jugal

The jugal in *Caiuajara* is a tetraradiate element [24]. Only the left jugal is preserved. The anterior portion is slightly displaced within the nasoantorbital fenestra with the ascending process and part of the anterior process exposed. The ascending lacrimal process of the jugal is not articulated with the lacrimal. This process is thin and sub-vertically oriented extending dorsally about 12 mm. The left anterior process overlaps the maxillary lateral surface. The posterior portion is ventrally displaced.

#### Lacrimal

The right lacrimal is medially displaced within the nasoantorbital fenestra. It is rotated 180°. The ventral half is partially missing. It is roughly triangular and highly fenestrated with a preserved height of 12 mm. There is also an elongated bone fragment overlapping part of the nasal. This element could be part of the lacrimal because of its elongated and highly fenestrated shape.

#### Quadrate

The right quadrate is missing. The mandibular condyle of the left quadrate is still connected to the articular. Anteriorly, it can be recognized by a large pterygoid process of the quadrate with 7.1 mm across. The posterior portion of the quadrate is partially covered by the matrix. The element is medially slender extending approximately 40 mm with rounded tips. Its posterior side connects to opisthotic. It is not well preserved.

#### Occipital

**Fig 7:**
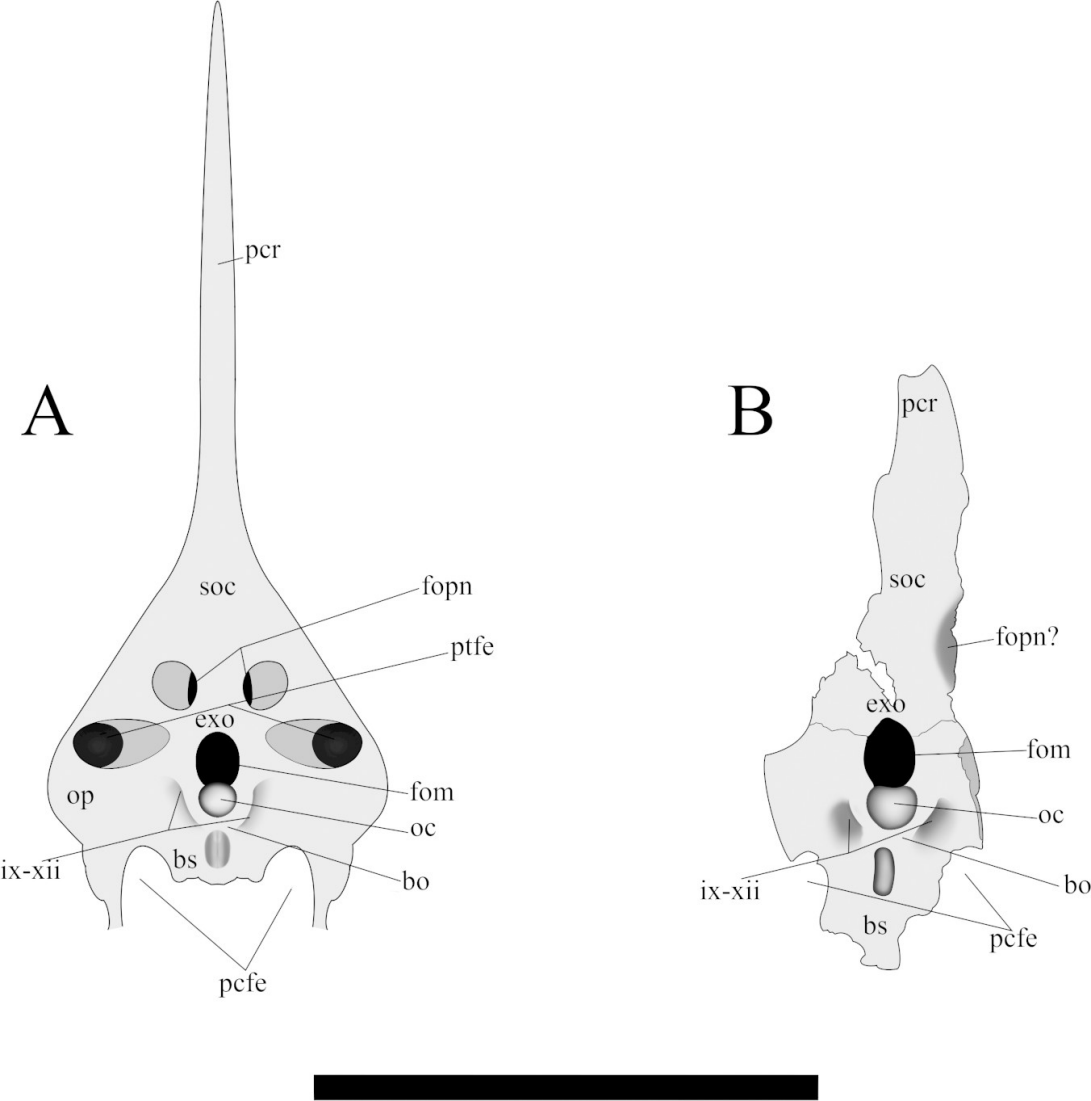
Schematic drawing of the occipital region of *Caiuajara dobruskii* in ventral view. (A) specimen CP.V 8175, (B) specimen CP.V 1449. Scale bar equals 50 mm.

The right occipital region is covered by the matrix. All the occiput is concave and posterolaterally perforated by the left post temporal fenestra (Fig 7A). Slightly posterior to the opening of the post temporal, the occiput is pierced by a large pneumatic foramen. The widest region of the occiput is located on the level of the post temporal fenestra, tapering posteriorly. The occipital condyle is well-developed with a rounded ventral surface. The basisphenoid preserves its posterior portion. It presents a knob-like protrusion anteriorly to the occipital condyle and indicates the medial border of the postcranial fenestra. In the joint between opisthotic and basioccipital, laterally to the occipital condyle is present two concavities referent to the passage of the cranial nerves IX-XII.

#### Supraoccipital

The supraoccipital is partially covered on the right side. This skeletal element comprises the upper portion of the foramen magnum and the lateral border of the postemporal fenestra. The supraoccipital articulates with the exoccipital through a slender and smooth suture. Posteriorly to the postemporal fenestra, the supraoccipital is pierced by a large pneumatic foramen. Posteriorly the bone tapers and comprises the ventral surface of the parietal crest.

#### Exoccipital, opisthotic, and basioccipital

The exoccipital delineates most of the lateral border of the foramen magnum, but its contact with opisthotic is not visible. The basioccipital comprises the occipital condyle.

#### Basisphenoid

The basisphenoid is only posteriorly preserved, delimiting the posterior medial margin of postcranial fenestra. Such element is at least 8.8 mm wide. Ventrally, the basioccipital and basisphenoid are fused without a recognizable suture. It should be noted that an elongated and large bone displaced medially at the skull could correspond to the anterior portion of the basisphenoid. However, this long bone seems to be considerably narrower than the posterior preserved portion of the basisphenoid in CP.V 8175. Furthermore, the weathering of this narrow bone and its anterior displacement raise ambiguities on its accurate identification.

#### Pseudomesethmoid

The pseudomesethmoid could be identified as an ossified structure inserted between the medial part of the frontals, forming the interorbital septum. Posteriorly, this element exhibits a large opening for passage of the optic nerve (II) followed ventrally by the opening for cranial nerves.

#### Hyoid apparatus

Fragments of hyoid apparatus are spread all over the ventral portion of the skull. These are slightly curved, slender, and rod-like bones. Between the dentaries is preserved an element that can be associated with the left ceratobranchial.

#### Lower jaw

**Fig 8:**
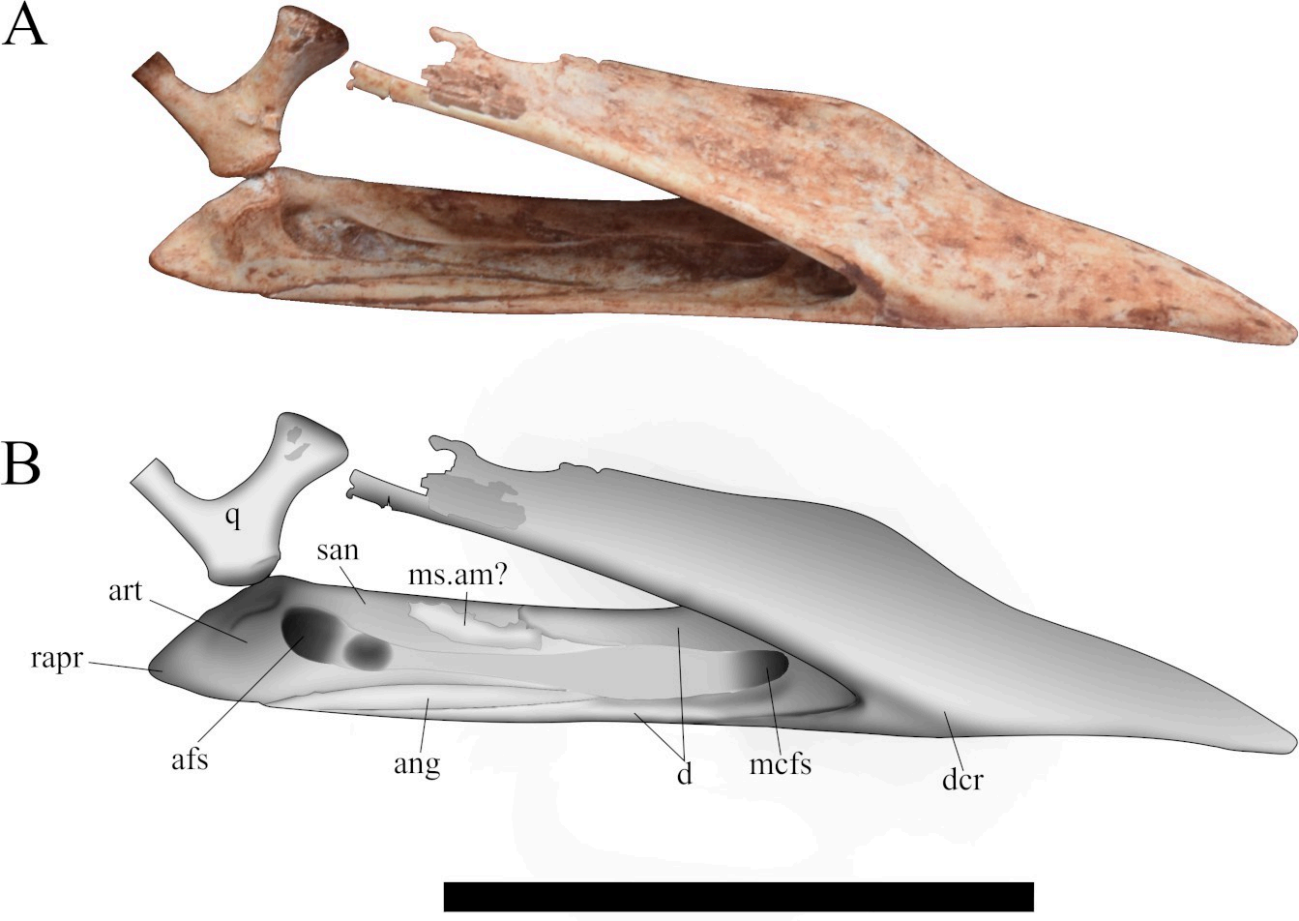
Mandibular ramus of the specimen CP.V 8175 in lateral view. (A) Picture; (B) Schematic drawing. Scale bar equals 50 mm.

As in all azhdarchoids, the lower jaw is toothless [43, 44]. It is laterally compressed with only limited damage. The right mandibular ramus is broken posteriorly. This ramus is displaced dorsoposteriorly to the symphyseal. The left dentary ramus is complete, exposing only one lingual surface (Fig 8), and it is still connected to the quadrate condyle. The dentaries are fused forming the mandibular symphysis with no visible suture. The total jaw length is 2.6 (ML/SL) time larger than the symphysis length. The anterior tip of the lower jaw is inclined downwards. Dorsally, at the same level of the dentary crest, the occlusal surface bears a depression exposing a concavity. Such concavity matches with the ventral protrusions of the upper jaw during occlusion.

The left mandibular ramus is composed by the dentary, the surangular, the angular, and the articular, with the absence of the splenial. In the anterior portion of the mandibular ramus, the anterior margin of the Meckelian fossa can be identified. Posteriorly, the Meckelian fossa is confluent with the adductor fossa extending through almost all of the length of the ramus. This particular shape can be seen due to the absence of the splenial. The adductor fossa is deeply excavated, starting posterior to the symphysis and extending almost to the level of the retroarticular process. On its posterior portion, the adductor fossa shows an oval depression. It extends from the most anterior tip to the middle portion of the lower jaw. On the lingual side of the ramus, the dentary overlaps the surangular dorsally and contacts the articular medially. Ventrally, it articulates with the angular and tapers gently forming the majority of the ventral margin of the ramus posterior to the symphysis.

The surangular is overlapped by the dentary anteriorly, and contacts the articular posteroventrally with no visible sutures. This element exhibits a depression on its middle portion, which could be the insertion of muscle adductor mandibulae. On the posterior portion of the ramus, it comprises the upper margin of the adductor fossa. In lateral view, this bone is slightly arched forming the ramus dorsal portion. It has an approximate length of 26.1 mm, the absence of a posterior suture inhibits a more precise measure.

The angular in *Caiuajara* is reduced and splint-like, forming a small portion of the medial surface of the left ramus and extending posteriorly up to its ventral margin. Its maximum length is 28.5 mm. Anteroventrally, the angular is overlapped by the dentary, whilst the articular lies over it posterodorsally.

The articular is a large bone that participates in the medial surface and most posterior portion of the ramus. It comprises the majority of the adductor fossa posterior portion and retroarticular process, contacting the surangular anteriorly with no visible suture. The articular overlaps the angular medially and articulates with the dentary anteriorly with the absence of the splenial. The glenoid fossa is covered by the quadrate. The upper surface of the retroarticular process slopes downward at an angle about 40° and slightly arches medially. In the right mandibular rami, the retroarticular process bears a rounded *fossa depressoria*, occupying most of its medial surface. This fossa is probably an attachment of the *M*. *depressor mandibulae*.

#### Additional observations based on other specimens of *Caiuajara dorbuskii*

The additional specimens used here to compare anatomic features have a significant scope in shape and size (Figs 2 and 3). Specimens CP.V 1050–1 and CP.V 8171 are interpreted here as juveniles due those dimensions. The holotype (CP.V 1449) is described as at least an older subadult individual [24]. Other specimens as CP.V 1005, CP.V 1448, and CP.V 5703, with exception of CP.V 5703 which are not well preserved, shows maturity features such as its size and inclined sagittal crest. But this interpretation needs to be handled with caution once only CP.V 1005 have a well preserved premaxillary crest and was previously interpreted as an adult individual by Manzig [24] (See Table 3).

**Table 3:** Comparison of the identified ontogenetic stages in the Tapejarinae specimens discussed in this work.

Species	Specimen	Ontogeny	Reference
*Caiuajara dobruskii*	CP.V 8175	Sub-adult	This article
*Caiuajara dobruskii*	CP.V 1449	Sub-adult/adult	Manzig et al. 2014
*Caiuajara dobruskii*	CP.V 8171	Juvenil	This article
*Caiuajara dobruskii*	CP.V 5703	At least sub-adult	This article
*Caiuajara dobruskii*	CP.V 1005	Adult	Manzig et al. 2014
*Caiuajara dobruskii*	CP.V 1448	Sub-adult/adult	This Article
*Caiuajara dobruskii*	CP.V 1050–1	Juvenil	Manzig et al. 2014
*Tupandactylus imperator*	MCT 1622-R	Adult	Campos & Kellner 1997
*Tupandactylus imperator*	CPCA 3590	Adult	Pinheiro et al. 2011
*Tupandactylus navigans*	GP/2E 9266	Adult	Beccari et al., 2021
*Tapejara wellnhoferi*	AMNH 24440	Juvenile/sub-adult	Wellnhofer & Kellner 1991
*‘Huaxiapterus’ benxiensis*	BXGM V0011	Adult	Junchang et al. 2007
*Sinopterus dongi*	IVPP V 13363	Sub-adult	Wang et al. 2003
*Sinopterus atavismus*	IVPP V 23388	Juvenile	Zhang et al. 2019

The premaxilla has a significant variation between individuals. Specimens CP.V 1005 and CP.V 1448 (Fig 2B and 2E) have a thicker posterodorsal margin of the sagittal crest if compared with CP.V 8175 (Fig 1). Such a thick margin creates a dorsal projection making an enlarged concave posterior margin of premaxillomaxilla.

**Fig 9:**
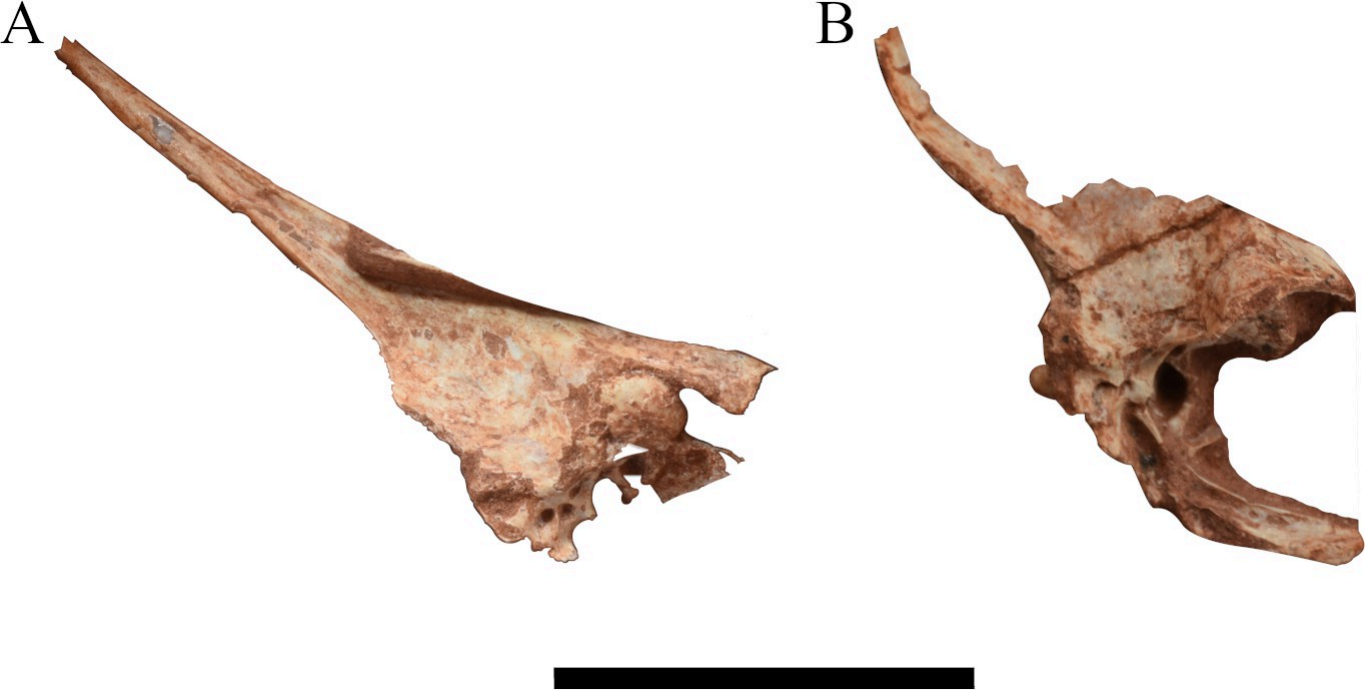
The parietal crest of *Caiuajara dobruskii*. (A) at the specimen CP.V 8175 in right lateral view; (B) at the specimen CP.V 1449 (holotype) in right lateral view. Scale bar equals 50 mm.

A slightly dorsal projection in the middle portion of the premaxillary posterior process exhibited by specimen CP.V 8175 are observed in three other specimens with variable sizes CP.V 1449, CP.V 1050–1, and CP.V 5703 (Fig 2A, 2C and 2F). Regarding the occiput, it is partially preserved in specimen CP.V 1449 (Fig 7B). However, the parietal crest is weathered on the lateral faces, and its most posterior portion is missing. Indeed, only the medial border of the post temporal fenestra is preserved in CP.V 1449. The basisphenoid preserves its posterior portion. The parietal process tapers posteriorly and has a slightly dorsal curvature (Fig 9B). The suture between supraoccipital and exoccipital runs almost horizontally in the half portion of the foramen magnum. The basisphenoid delineates the medial border of the postcranial fenestra, exhibiting a sharper protruded bone anterior to the occipital condyle, which apparently differs from that of CP.V 8175. Laterally, there is a depression on each side of the condyle with a rough texture, which may be for the passage of cranial nerves IX-XII.

**Fig 10:**
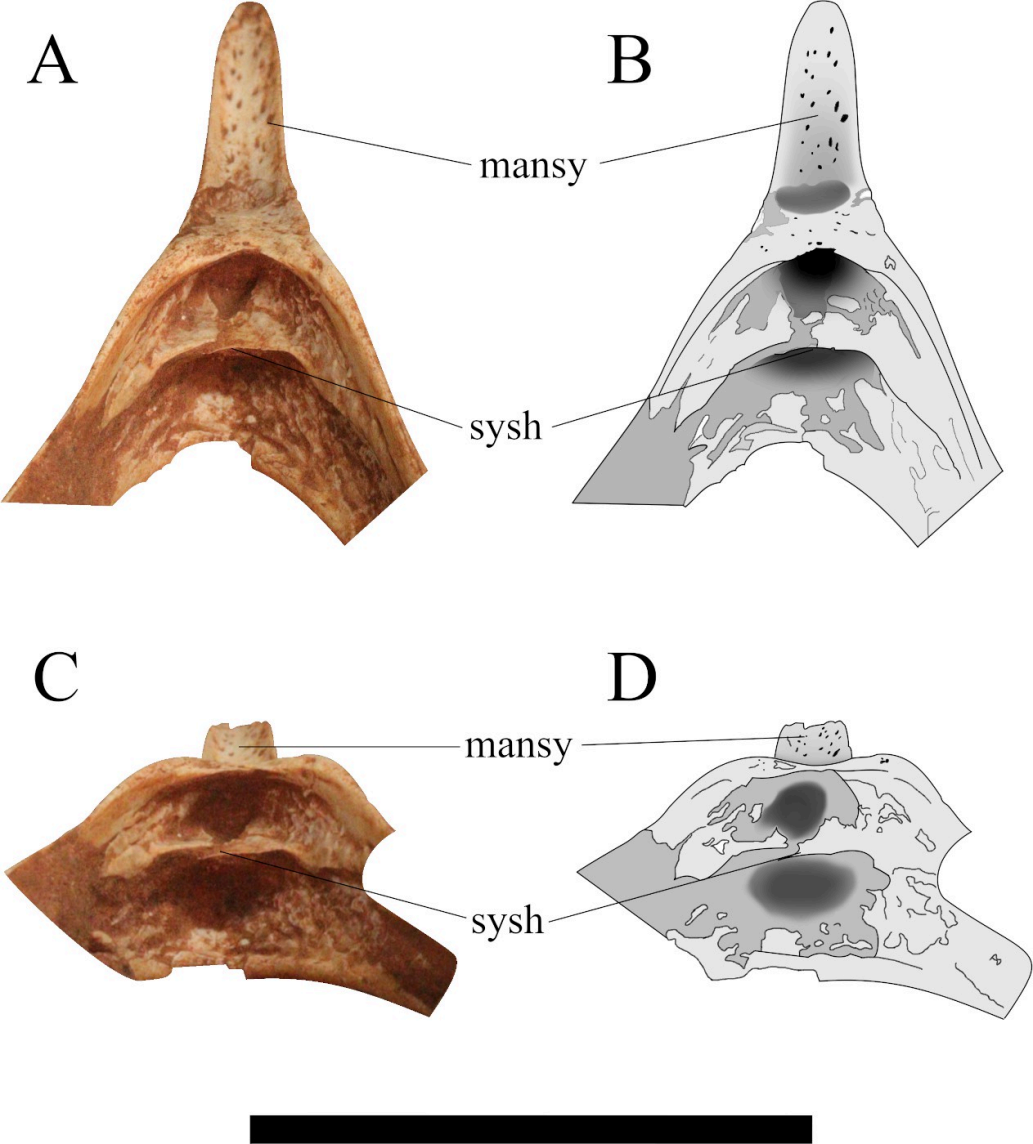
The mandibular symphysis of *Caiuajara dobruskii*. (A) Picture in posterodorsal view; (B) schematic drawing in posterodorsal view; (C) picture in posterior view; (D) schematic drawing in posterior view. Shaded patches are marking concavities as a result of the suture processes of the symphyseal shelf. Scale bar equals 50 mm.

Specimen CP.V 5703 also comprises an almost complete lower jaw (Fig 10), which presents several fractures scattered all over it. The left mandibular ramus is posteriorly weathered, whilst its right ramus is covered by other cranial elements. The symphysis comprises about one-third of the lower jaw total length, with no visible sutures. The dentaries merge at three different points. The first occurs dorsally at the occlusal region. The second ventrally at the dentary crest. And the last between those two. This condition creates two openings internally in the symphyseal, forming a shelf between the dorsal and ventral fusion (Fig 10B and 10D).

## Discussion

The original description of *Caiuajara dobruskii* was based on incomplete skulls, some of which lacked information on the relative positions of the posterior elements and did not properly describe the mandible.

The lack of a suture between the maxilla and premaxilla is a common feature among tapejarine pterosaurs [39, 45–54]. The rostral value (RV) *sensu* [41] of the specimen CP.V 8175 is similar to that of *Tupandactylus navigans* (GP/2E 9266) [54]. The first description of *Caiuajara* did mention the presence of a palatal ridge as a faint longitudinal crest [24] (Fig 4), a feature shared with *Tupuxuara* and *Thalassodromeus sethi*. Such structure is absent in *Caupedactylus ybaka*, *Tapejara wellnhoferi*, and hitherto was not reported for *Tupandactylus* [45, 51, 54, 55]. An irregular palatal surface is also seen in *Afrotapejara zouhrii* [56]. The posterior palate expansion present in *Caiuajara* is also distributed throughout the clade Tapejarinae as *Tapejara wellnhoferi*, *Caupedactylus ybaka*, or *Sinopterus dongi* [45, 48, 53, 55].

Regarding the size and inclination of the premaxillary sagittal crest have been interpreted as an ontogenetic variation [24]. In this way, adult individuals have larger and steeper (~90°) crests. Otherwise, the juveniles exhibit smaller and inclined (~115°) premaxillary sagittal crests. As the main case-study in this work, we note that CP.V 8175 exhibits a roughly similar size regarding the premaxillary crest to the holotype (CP.V 1449), described as an adult individual, even though it is a little smaller, with a more inclined premaxillary sagittal crest (Fig 3). In this way, CP.V 8175 is larger than CP.V 1050–1 and CP.V 8171, showing a steeper and more developed crest. All those features might be related to a subadult individual, which matches with the associated fused carpals.

The specimens CP.V 1449 (Fig 2A), CP.V 1050–1 (Fig 2C), CP.V 8171 (Fig 2D), and CP.V 5703 (Fig 2F) preserve part of the premaxillary posterior process. With exception of CP.V 8171, such aforementioned specimens present a slightly dorsal projection in the middle portion of its premaxillary posterior process. This feature was not previously reported, even by Manzig [24]. In particular, CP.V 8171 does not show such projection presumably due to the degree of weathering in this region. The specimens CP.V 8171 (Fig 2D) and CP.V 8175 (Fig 1) represent two different-sized individuals, both displaying the full extension of the posterior process of the premaxilla. Such a process was not reported with those dimensions in the first description of the species due the lack of preservation, which could possibly implicate in the general recognition of their premaxillary sagittal crest in variable size individuals. Furthermore, specimens CP.V 1005 (Fig 2B) and CP.V 1448 (Fig 2E) possess a thickened posterodorsal margin of the premaxillary sagittal crest, exhibiting a distinct morphology: in lateral view could be observed a gently rounded and oval margin of the premaxillomaxilar above the nasoantorbital fenestra. This condition differs from CP.V 1447 (Fig 3) and CP.V 8175 (Fig 1), both which have a similar size to CP.V 1448 (Fig 2E). That ought to indicate at least two different morphotypes regarding the premaxillary sagittal crest, which might be interpreted as sexual dimorphism, such as it has been previously interpreted in other pterodactyloid species with several specimens found together [25, 57]. But this premise must be handled with wariness once individuals with not fully developed features can resemble an adult of the opposite sex. As seems in young males of *Gavialis gangeticus* that looks like adult females [58], in this case such a trait could simple not been totally developed yet. On the other hand, even if individuals of the same species can display features at different degrees [58], is expected some traces of aspects that makes part of a continuous growth in similar sizes individuals as seen in *Casuariaus casuarius*, which presents a bony casque that grows continuous though ontogeny [59]. In this bird model is seen a graduated transition between not fully developed casque in immature individuals through complete casque in adults. Despite of that, point out sexual dimorphism in extinct groups is not an easy task unless there are a very clear pattern between the sexes, even in modern *Gavialis* continuous variables do not show much without previously known the gender [58].

### Comments on the morphological variation of the parietal process in Tapejarinae

**Fig 11:**
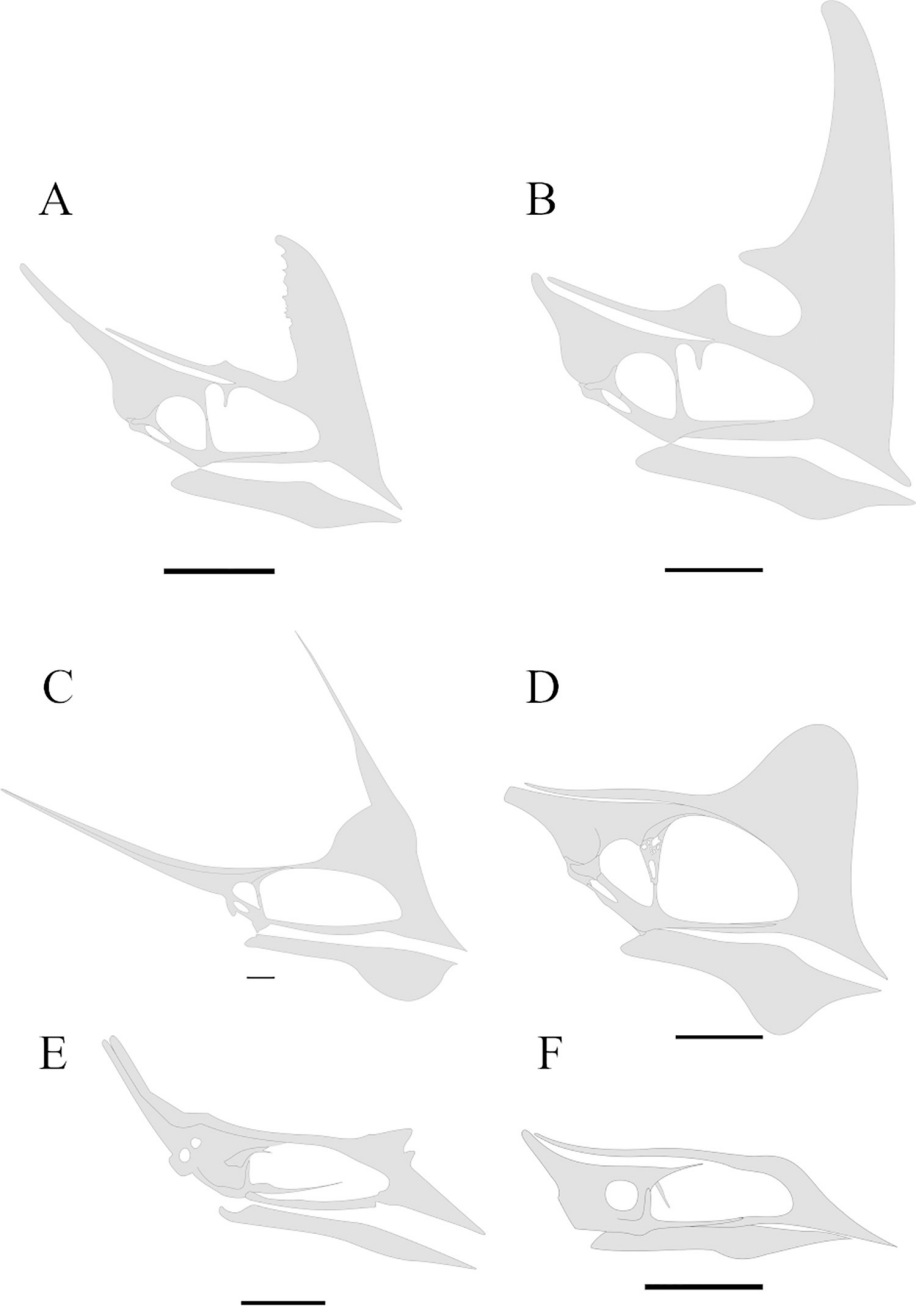
Silhouette of the crania of some Tapejarines pterosaurs. (A) *Caiuajara dobruskii* Specimen CP.V 8175; (B) the holotype of *Caiuajara dobruskii* CP.V 1449; (C) basic interpretation of *Tupandactyus imperator* MCT 1622-R; (D) *Tapejara wellnhoferi* AMNH 24440, modified from Wellnhofer & Kellner 1991; (E) *‘Huaxiapterus’ benxiensis* BXGM V0011); (F) *Sinopterus dongi* IVPP V 13363 modified from Wang & Zhou 2003. Scale bar equals 50 mm.

The parietal process in the specimen CP.V 8175 (Fig 1) increases the extension of the sagittal crest posteriorly, modifying the original interpretation of the sagittal crest in *Caiuajara* by Manzig [24]. Based on those new specimens, at least in some individuals this crest seems to extend more posteriorly than dorsally. Specimens CP.V 8175 (Fig 1) and CP.V 1449 (Fig 2A) exhibit differences in shape and extension of the parietal process (Fig 9). The dorsal curvature exhibited by CP.V 1449 differs from the straight extended condition observed in CP.V 8175. It is unlikely that those distinct morphologies of the parietal processes might be exclusively related to ontogeny due to the degree of development in each specimen: even though CP.V 8175 seems to be a bit younger than CP.V 1449, as suggested by the several sutures between the cranial elements, as those from the supraoccipital to exoccipital (for further details, see below regarding the occipital region). A possible problem for this assumption is that similar sized or aged individuals could express features in different degrees as seen in *Gavialis gangeticus* [58]. But it is unlikely that the parietal crest of CP.V 1449 would reach the proportion seems in CP.V 8175, in addition CP.V 1449 is already an older individual. Regarding other tapejarine species, such a process displays a high morphological variability, even though of ambiguous ontogenetic stages. *Tupandactylus imperator* shows a straight and well-developed parietal process (Fig 11C), similar to that of the specimen CP.V 8175 (Fig 11A) interpreted here as a subadult individual. *Tapejara wellnhoferi* exhibits a short parietal process which not seems to grown more than what is seems in the specimen AMNH 24440 (Fig 11D), similar to CP.V 1449 (Fig 11B) which is possible an adult individual of *Caiuajara*. Meanwhile *Tupandactylus navigans* exbibit an extremely reduced and rounded parietal process (Beccari et al. 2021) (Table 4). Other pterosaurs as ‘*Huaxiapterus*’ *benxiensis* (BXGM V0011), exhibit a well-developed and dorsally curved parietal process (Fig 11E), which is significantly larger than the specimen of *Sinopterus dongi* (IVPP V 13363), that shows a straight and less developed process (Fig 11F). Naish [60] hypothesises that both ‘*H*.’ *benxiensis* and *S*. *dongi* are synonyms, assuming BXGM V0011 (‘*H*.’ *benxiensis*) is a mature individual of *S*. *dongi*. However, such presumption should be treated with caution due to lack of information regarding the stratigraphy and geochronology of Jiufotang Formation outcrops. Thus, the species could not be synchronic [53, 61].

**Table 4:** Comparison of both morphology of the parietal crest and contact of the premaxilla with the dorsal margin of the skull in the Tapejarinae specimens discussed in this work. The specimens compared are those Tapejarinae with at least preserved one of those features analysed.

Species	Specimen	Parietal crest	Contact of the premaxilla
*Caiuajara dobruskii*	CP.V 8175	Straight and well-developed	Only nasals
*Caiuajara dorbuskii*	CP.V 1449	Curved and short	Not preserved
*Tupandactylus imperator*	CPCA 3590	Straight and well-developed	Nasals and frontoparietal
*Tupandactylus navigans*	GP/2E 9266	Highly reduced and rounded	Nasals and frontoparietal
*Tapejara wellnhoferi*	AMNH 24440	Short	Absent
*Sinopterus dongi*	IVPP V 13363	Straight and short	Absent
*‘Huaxiapterus’ benxiensis*	BXGM V0011	Curved and well-developed	Not preserved
*Sinopterus atavismus*	IVPP V 23388	Not preserved	Absent

### Comparative approach of the occipital region

Regarding the occipital region, CP.V 8175 (Fig 7A) and CP.V 1449 (Fig 7B) are the only specimens of *Caiuajara* currently known which preserve this. In particular, the sutures between the supraoccipital and the exoccipital are clearly more visible in the holotype (CP.V 1449), that is probably due to a state of incomplete ossification between those elements in specimen CP.V 8175, which may cause a unclearness of those facets. A similar case is reported for *Bellubrunnus rothgaengeri* [62], where some skull sutures seem to be incomplete probably due to the immaturity of the individual. Such a fact might suggest a more developed state of maturity of specimen CP.V 1449. *Anhanguera piscator* [63], and *Pteranodon longiceps* [64] shows a similar pattern of fusion, as well as specimen MCT 1500-R, which may correspond to *Tapejara wellnhoferi* [65]. The supraoccipital extension and the lateral expansion are forming most of the upper portion of the occiput [65] in CP.V 8175, a condition which is widely present within the clade Ornithocheiroidea [16, 29, 43, 44, 57, 66]. This condition differs from early-diverged pterosaurs, in which the supraoccipital is comparatively small [19, 43, 67]. In addition, all known members of the Pterodactyloidea display a pneumatic foramen and post temporal fenestra in the supraoccipital [65] as shown in the new specimen. The presence of the posterior portion of the basisphenoid allows identifying the location of the postcranial fenestra and asserts the elongated and anteroventrally directed condition widely spread among pterosaurs [17, 28, 55, 63].

### On the variability of the contact between premaxilla and frontoparietal in Tapejarinae

Within the Tapejarinae, various species exhibit different conditions in the contact between the premaxilla and dorsal margin of the skull formed by the frontoparietal. Discussions related to the different conditions found throughout species are recurrent [48, 51]. And it is not clear whether the fusion between those bones is progressively developed throughout ontogeny or it could disarticulate during the fossilization process.

In this way, in *Tupandactylus imperator* (CPCA 3590) and *Tupandactylus navigans* (GP/2E 9266) the premaxilla is contacting the nasals and the frontoparietal (see [51, 54]). Whereas in *Tapejara wellnhoferi* (AMNH 24440), *Sinopterus dongi* (IVPP V 13363), and *Sinopterus atavismus* (IVPP V 23388) such contact is absent [45, 48, 53] (See Tables 3 and 4). CP.V 8175 (Fig 5) of *Caiuajara dobruskii* displays a contact between premaxilla and nasals, but it is absent through the frontoparietal. Thus, it seems to be a trend within Tapejarinae where this contact would be observed only in fully developed individuals known so far, as in specimen CPCA 3590 of *Tupandactylus imperator* and in specimen GP/2E 9266 of *Tupandactylus navigans*. Between the different *Caiuajara* specimens analysed here, only CP.V 8175 preserves such a structure, displaying a completely different condition. That could indicate either adulthood had not been reached or even an interspecific feature within Tapejarinae, but the latter assumption might be elucidated with new finding belonging to *Caiuajara dobruskii* (or even other Tapejarini species) preserving such structure in a more complete ontogenetic series.

### Morphological significance and considerations in *Caiuajara dobruskii* lower jaw

Regarding the lower jaw of *Caiuajara*, the description of CP.V 8175 (Fig 8) and CP.V 5703 (Fig 10) allows an accurate understanding of its morphology. From those autopomorphies used by Manzig [24] to diagnose *Caiuajara dobruskii*, just one was related to the lower jaw: a rounded depression in the occlusal concavity of the dentary [24]. The dentaries are sutured together at three different points as exhibited by CP.V 5703, which seems to be a unique feature among known pterosaurs. Although not so well-preserved, this condition is also present in specimens CP.V 8175 and CP.V 1449. Therefore, this unique feature can be considered as a new autapomorphy of *Caiuajara dobruskii*. Furthermore, CP.V 8175 displays an extremely elongated adductor fossa (Fig 8) as described in this work. Such conditions can be related to the absence of the splenial, which in other pterosaurs is displaced in the middle portion of the mandibular ramus as in *Anhanguera piscator*, and *Thalassodromeus sethi*.

## Conclusion

**Fig 12:**
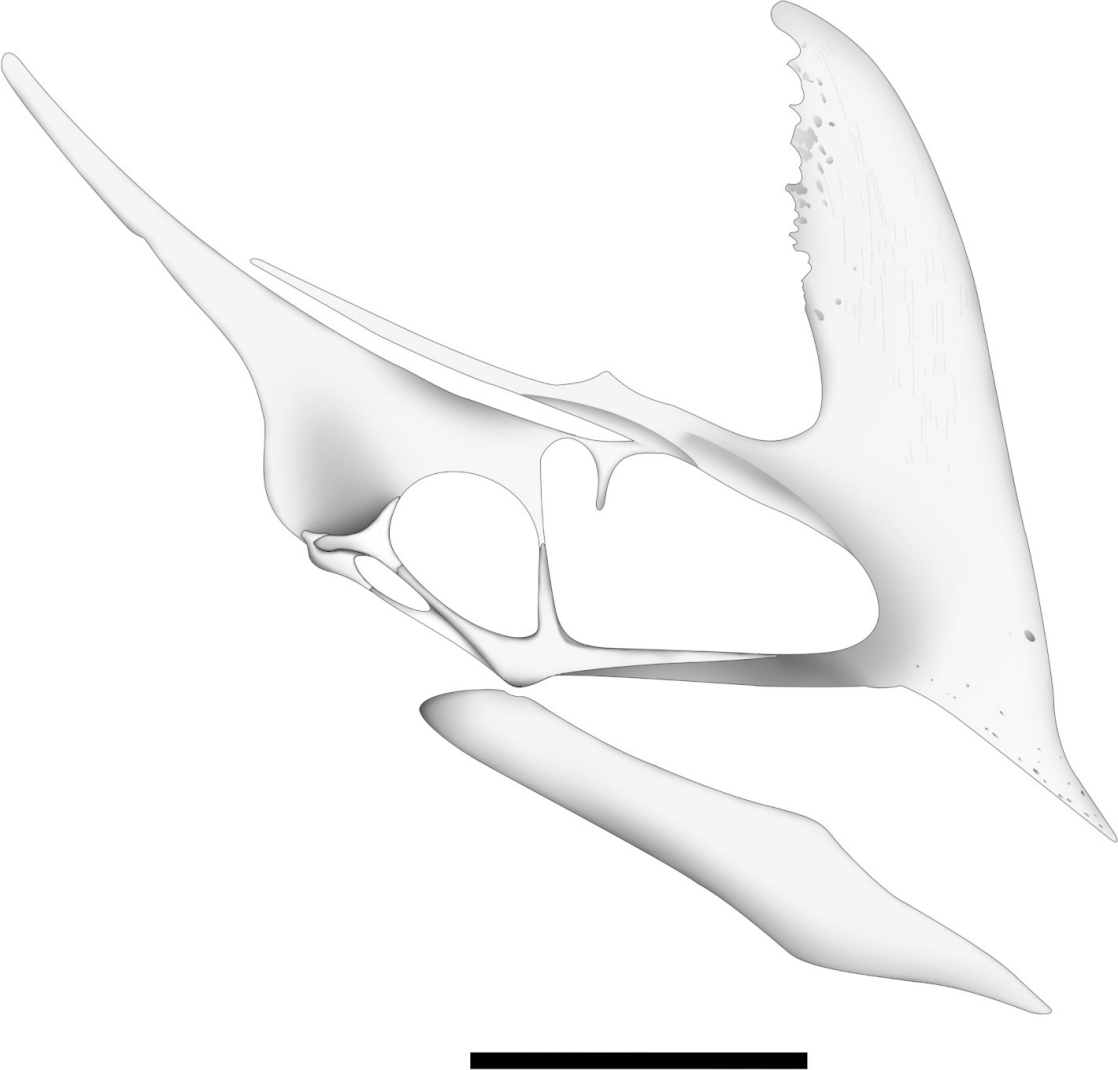
Interpretation of specimen CP.V 8175 skull on the basis of the preserved cranial elements and comparison with other tapejarins. Scale bar equals 50 mm.

The new specimens of *Caiuajara dobruskii* described in this article provided new essential insights related to cranial anatomy and morphological variability. This new material revealed several anatomical traits which allow a comprehensive understanding of the cranial anatomy in *Caiuajara dobruskii*, including the morphology of the sagittal crest (Fig 12). We also provide information related to the basicranium of *Caiuajara*, enabling a new interpretation of the occipital region, including the posterior limit of the postcranial fenestra. We describe a well-developed occipital condyle, the presence of a pneumatic foramen in the supraoccipital, and the suture of particular elements assembling that region. Likewise we also characterize the lower jaw of *Caiuajara* in detail, reporting an absence of the splenial, and featuring an extremely elongated adductor fossa. Furthermore, the dentaries merge at three different points, forming a symphysis shelf, this feature is proposed here as a new diagnosis for *Caiuajara dobruskii* once it is not identified in any other known pterosaur. In addition, we reported here variations between different specimens regarding the thickness of the posterodorsal margin of the premaxillary sagittal crest, which may be inferred as sexual dimorphism. Variation in the shape and extension of the parietal process is also noted. Such variability demonstrates intraspecific variations, and it was compared with some other tapejarines to help to elucidate some debate regarding its identification. Information concerning the skull and the lower jaw of *Caiuajara dobruskii* presented here provides key data for subsequent research.

## Supporting information

S1 Fig Isolated postcranial elements of the specimen CP.V 8175. (A) pteroid; (B) isolated manual phalanx; (C) ungueal phalanx; (D) proximal carpals; (E) left IV metacarpal in anterior view; (F) left IV metacarpal in posterior view.
(TIF)

S2 Fig Picture of the whole block of specimen CP.V 8175. (TIF)
