# Supplementary figures and images for: Novel information on the cranial anatomy of the tapejarine pterosaur Caiuajara dobruskii
Source: PLoS One. 2022 Dec 15;17(12):e0277780. doi: 10.1371/journal.pone.0277780 (PMC9754175; doi:10.1371/journal.pone.0277780)

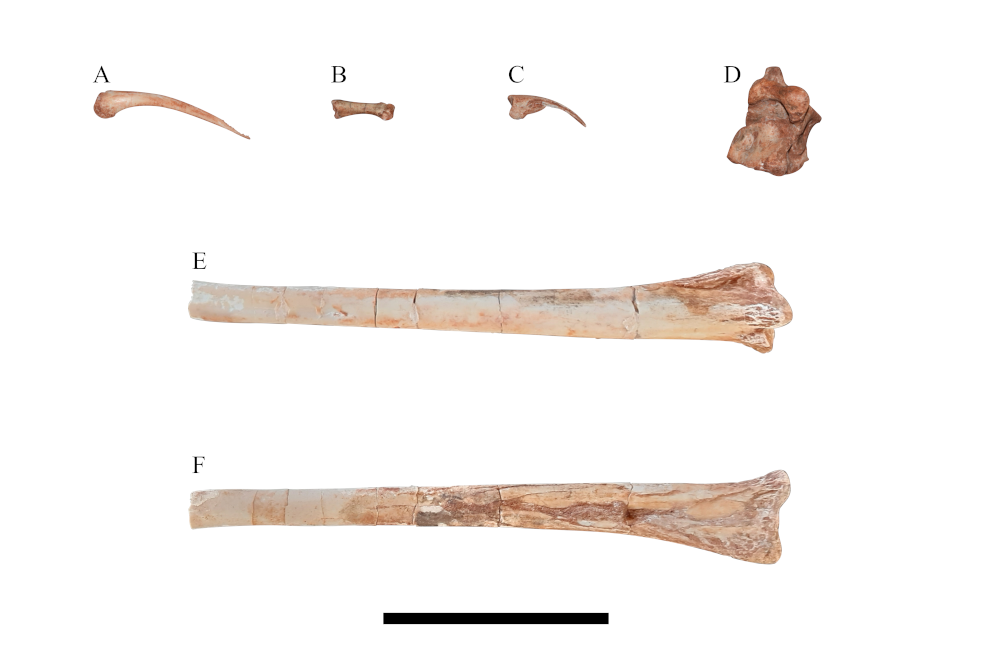

Supplement: S1 Fig — (A) pteroid; (B) isolated manual phalanx; (C) ungueal phalanx; (D) proximal carpals; (E) left IV metacarpal in anterior view; (F) left IV metacarpal in posterior view. (TIF) [file pone.0277780.s001.tif]

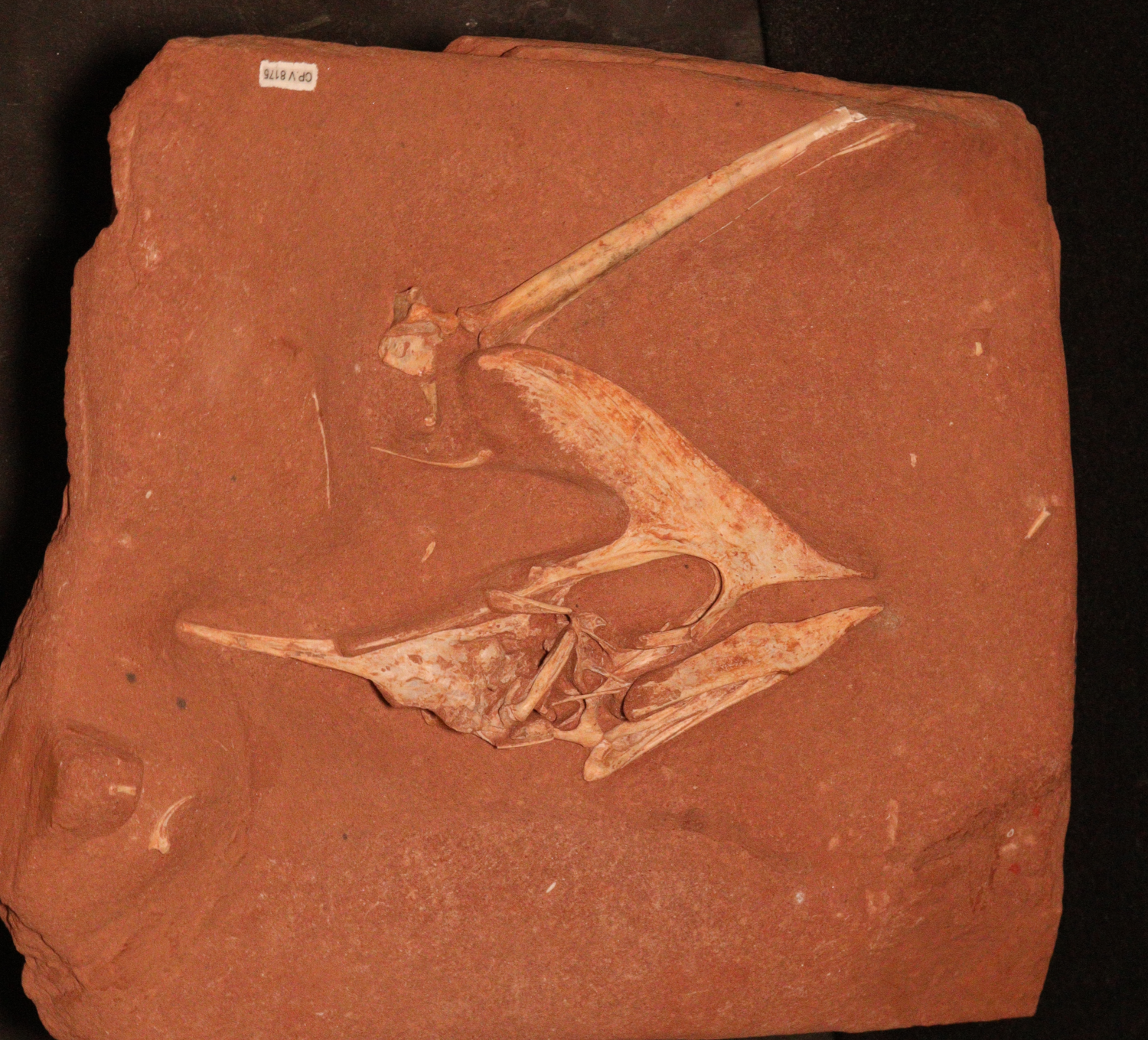

Supplement: S2 Fig — (TIF) [file pone.0277780.s002.tif]
